# Combinational Immunotherapy for Hepatocellular Carcinoma: Radiotherapy, Immune Checkpoint Blockade and Beyond

**DOI:** 10.3389/fimmu.2020.568759

**Published:** 2020-09-30

**Authors:** Yun Hua Lee, David Tai, Connie Yip, Su Pin Choo, Valerie Chew

**Affiliations:** ^1^ Translational Immunology Institute (TII), SingHealth-DukeNUS Academic Medical Centre, Singapore, Singapore; ^2^ Division of Medical Oncology, National Cancer Centre, Singapore, Singapore; ^3^ Division of Radiation Oncology, National Cancer Centre, Singapore, Singapore; ^4^ Curie Oncology, Mount Elizabeth Novena Specialist Centre, Singapore, Singapore

**Keywords:** radiotherapy, immunotherapy, immune checkpoint blockade (ICB), combination therapy, hepatocellular carcinoma (HCC)

## Abstract

The systemic treatment landscape for advanced hepatocellular carcinoma (HCC) has experienced tremendous paradigm shift towards targeting tumor microenvironment (TME) following recent trials utilizing immune checkpoint blockade (ICB). However, limited success of ICB as monotherapy mandates the evaluation of combination strategies incorporating immunotherapy for improved clinical efficacy. Radiotherapy (RT) is an integral component in treatment of solid cancers, including HCC. Radiation mediates localized tumor killing and TME modification, thereby potentiating the action of ICB. Several preclinical and clinical studies have explored the efficacy of combining RT and ICB in HCC with promising outcomes. Greater efforts are required in discovery and understanding of novel combination strategies to maximize clinical benefit with tolerable adverse effects. This current review provides a comprehensive assessment of RT and ICB in HCC, their respective impact on TME, the rationale for their synergistic combination, as well as the current potential biomarkers available to predict clinical response. We also speculate on novel future strategies to further enhance the efficacy of this combination.

## Introduction and Background on Current Treatment Landscape for HCC

Liver cancer ranks second as the leading cause of cancer deaths in men and fourth highest cancer mortality in both genders globally with an estimate of 781,631 deaths in 2018 (1). Hepatocellular carcinoma (HCC), which comprises 75–85% of liver cancer cases, is a complex cancer with various etiologies such as hepatitis viral infection, alcohol abuse, and obesity (2). Chronic viral infection remains the primary contributing factor of liver cirrhosis, a chronic liver disease that precedes HCC, characterized by the irreversible scarring and hardening of the liver tissue with decreased hepatocyte proliferation (3, 4).The chronic liver inflammation causes fibrous scar tissues to replace healthy tissues overtime and eventually occlude blood flow through the liver, resulting in the onset of portal hypertension and creates a hypoxic environment that favors tumor growth (5–7). Cirrhosis also leads to impairment of the immune surveillance within the liver through the reduced synthesis of innate immunity proteins and pattern-recognition receptors (PRRs). This in turn damages the antigen-presenting and phagocytic capacity of Kupffer cells and sinusoidal endothelial cells which creates an immuno-deficient environment in patients and contributes to hepatocarcinogenesis (8, 9).

The management of early stage HCC with curative intent includes surgical resection or liver transplantation with expected 5-year survival rate between 60 and 80% (10). However, less than 20% of HCC patients qualify for these curative treatments (11). Loco-regional therapies such as radiofrequency ablation, chemo, or radio-embolization are recommended for local disease control when curative treatments are contraindicated (12). For unresectable advanced HCC, systemic therapies such as tyrosine kinase inhibitors (TKI) (e.g. Sorafenib) have been used (13). Sorafenib was approved by the Food and Drug Administration (FDA) as first-line therapy for inoperable HCC a decade ago and remains the current standard of care despite its relatively modest activity (14, 15). Lenvatinib, another first-line treatment option for advanced HCC, demonstrated non-inferiority in terms of median overall survival (OS), but superiority in progression-free survival (PFS) and overall response rate (ORR) compared to sorafenib (16). Regorafenib, cabozantinib, and ramucirumab have also attained FDA approval in recent years as second-line treatments for patients with advanced HCC who exhibited progressive disease after one or more systemic therapies (12).

More recently, immune checkpoint blockade (ICB) has emerged as a promising therapeutic option for advanced HCC patients (17). Both phase II and phase III ICB trials in advanced HCC using antibodies against programmed cell death protein 1 (PD-1) have demonstrated clinical benefit and fewer incidences of serious treatment-related adverse events (17–20). The recent IMbrave150 trial, which investigated the combination of atezolizumab (anti-PD-L1) and bevacizumab (anti-VEGF-A; anti-angiogenesis agent) in patients with advanced HCC, showed survival benefit over sorafenib in the first-line setting and highlighted the potential of combining ICB with a tumor microenvironment (TME)-modifying agent (21). In March 2020, FDA also approved the use of ipilimumab (anti-CTLA-4) combined with nivolumab (anti-PD-1) in sorafenib-experienced patients based on the high objective response rates (RR) and durability of responses (19).

Besides combinations of multiple ICBs or ICB with targeted therapies, growing evidence indicates the role of radiotherapy (RT) in potentiating tumor immunity (22, 23). For instance, preclinical studies have shown that combination of RT and ICB can synergistically augment the anti-tumor responses induced by both agents (24–26). In this systemic review, we will discuss the impact of RT and ICB on the TME and how this combinational therapy, and potentially along with another therapeutic agent, could bring about a synergistic control of HCC progression.

## Immune Landscape of HCC

**Figure 1:**
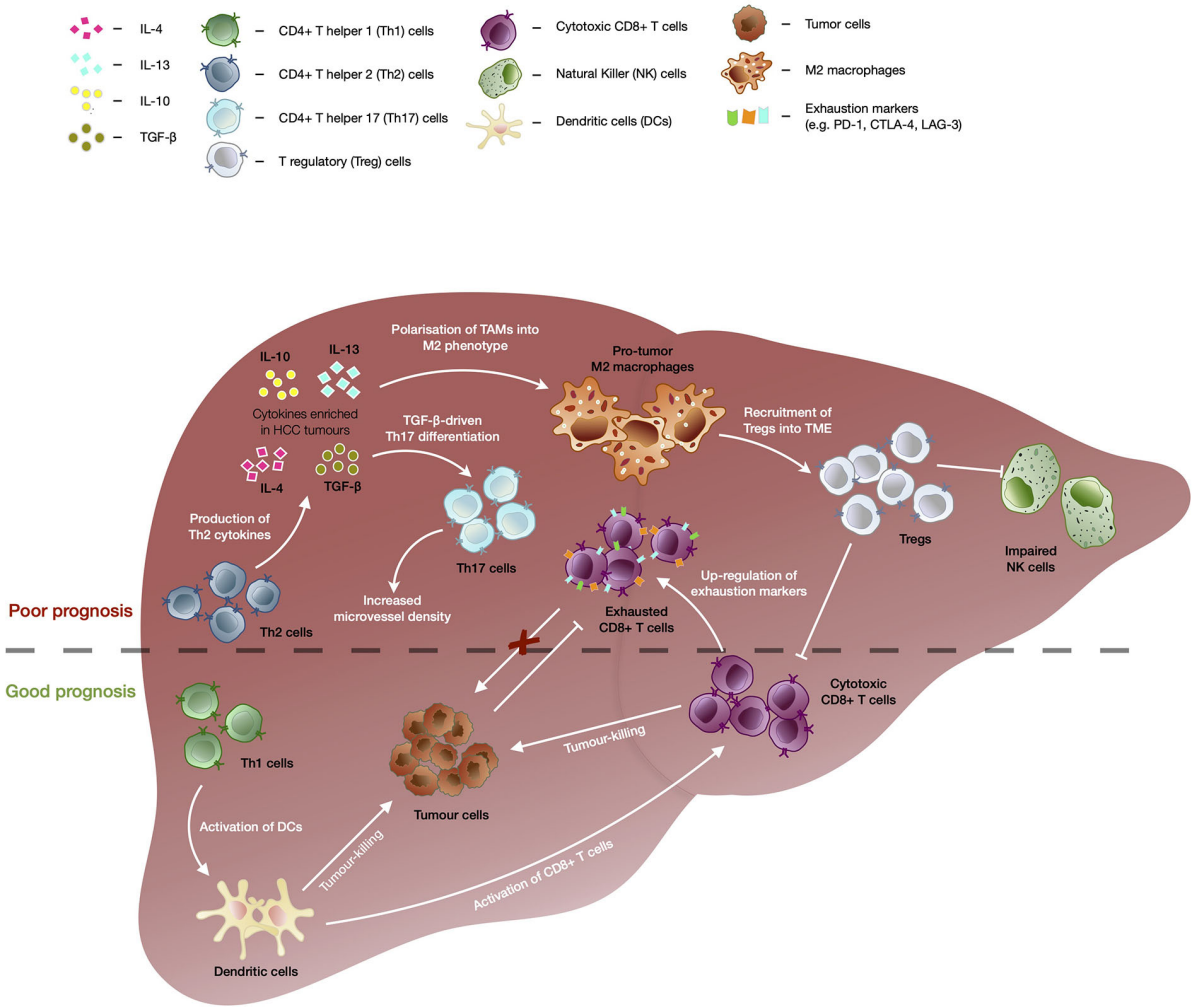
The immunological events contributing to good versus bad prognosis in HCC. Enrichment of CD4+ T helper 2 (Th2) cells are associated with poor overall survival of HCC patients. TGF-β as well as cytokines secreted by Th2 such as IL-4, IL-10, and IL-13, drives the polarization of tumor-associated macrophages (TAMs) into immunosuppressive M2 macrophage phenotype which further recruits T regulatory cells (Tregs). Tregs in turn further enhances the immunosuppressive tumor microenvironment through inhibition of CD8+ T memory/effector cells’ and natural killer (NK) cells’ tumor-killing capacity, leading to poor prognosis in HCC. TGF-β also drives differentiation of CD4+ T helper 17 (Th17) cells and high frequencies of Th17 have been correlated with increased microvascular invasion and shorter OS and DFS of HCC patients. Presence of CD4+ T helper 1 (Th1) cells correlated with favorable outcomes in HCC as they are able to enhance activation of CD8+ T cells *via* dendritic cells (DC) and trigger DC-mediated tumor-killing. Despite the capability to kill tumor cells and association with good prognosis in HCC, CD8+ T cells showed upregulation of exhaustion markers (e.g. PD-1, CTLA-4, and LAG-3), which dampens its killing capacity in chronic conditions leading to tumor progression.

Chronic hepatitis infection or long-standing liver injury often place the liver in a chronic inflammatory state (27). Modest inflammation confers protection against pathogens and repair tissue damage, however, sustained liver inflammation can perturb the microenvironment and tip the scales in favor of carcinogenesis (28–30). For instance, cytokines and chemokines secreted within the liver can promote angiogenesis, immune evasion and anti-apoptotic responses as well as to recruit immune cells with the capacity to create a niche microenvironment that facilitates tumor growth (31, 32). In particular, anti-inflammatory cytokine interleukin-10 (IL-10) was shown to be highly enriched in HCC tumors as compared to adjacent non-tumor or healthy liver tissues (33). High levels of transforming growth factor beta (TGF-β) was also associated with increased tumor invasiveness in advanced HCC (**Figure 1**) (34).

Tumor-associated cytokines and chemokines are able to recruit and polarize immune subsets into a pro-tumor phenotype and fuel tumorigenesis. One of such immune subsets is the tumor-associated macrophages (TAMs) which are polarized into M2 macrophage phenotype by IL-10, TGF-β, IL-4, or IL-13 present in the TME, and drive tumor progression by supporting angiogenesis and recruitment of CD4+FoxP3+ T regulatory cells (Tregs) (**Figure 1**) (35, 36). The accumulation of TAMs in the tumor region is frequently associated with poor prognosis across cancer types, including HCC (35, 37). In addition, Tregs which regulate or dampen CD8 T cells’ activation and cytotoxic capacity, also play a critical role in promoting tumorigenesis (38, 39). We previously reported that Tregs from HBV-positive HCC tumors exhibited higher expression of PD-1 and displayed superior suppressive capacity against CD8 T cells (30). Higher intra-tumoral interleukin-17-producing Tregs have also been consistently reported in HCC and correlated with poorer prognosis and reduced survival in HCC patients (30, 37, 38, 40, 41). Likewise, TGF-β-rich TME of HCC favours the differentiation of Th17, a CD4 T helper subset that also produces IL-17 (42). High intra-tumoral frequencies of Th17 were positively correlated with microvascular invasion in HCC patients and were implicated in shorter OS and disease free survival (DFS) of patients (43).

On the other hand, CD4 T helper 1 cells (Th1) that are capable of producing IFN-γ can activate dendritic cells which leads to enhanced priming and maturation of CD8 T cells (44, 45). Th1 can also trigger DC-mediated tumor-killing activities *via* IFN-γ-dependent mechanisms which will further fuel downstream anti-tumor immune responses (**Figure 1**) (46). An increase in Th1 response correlated with favorable outcomes in HCC patients following treatment with transarterial chemoembolization (TACE) (47). While Th1 demonstrated anti-tumor capabilities, type 2 CD4 T helper (Th2) were found to be enriched in HCC tissues as compared to normal healthy livers and were inversely associated with OS of HCC patients (48). Th2 cytokines such as IL-4, IL-10, and IL-13 are able to induce M2 macrophages which have reduced cytotoxic activity and dampens CD8 T cell-mediated anti-tumor activity (**Figure 1**) (49, 50). Furthermore, upregulation of Th2 cytokine production was linked to increased likelihood of HCV-induced HCC (51).

Cytotoxic immune populations such as CD8 T resident memory (Trm), CD8 T effector memory (Tem), and natural killer (NK) cells, play a pivotal role in anti-tumor immunity (37, 40). However, it was reported that NK cells found within the tumor regions of HCC tumors exhibited inferior cytolytic capacity and production of IFN-γ as compared to NK cells derived from non-tumor regions (52). The authors also observed that the addition of HCC patient-derived Tregs impaired the tumor-killing ability of autologous NK cells *in vitro* (**Figure 1**). While the presence of Trm and Tem cells was associated with good prognosis in HCC (30), they often express exhaustion markers such as PD-1, lymphocyte-activation gene 3 (LAG-3), and cytotoxic T-lymphocyte-associated antigen 4 (CTLA-4), which correlated negatively with their functional competency (**Figure 1**) (37, 53). Thus, these exhaustion markers have been the prime targets of ICB to reinvigorate and restore the cytotoxic capacity of CD8 T cells (39).

## Radiotherapy (RT) in HCC

RT for HCC was traditionally linked to suboptimal results due to limited tolerance of whole liver irradiation and the inability to conform radiation doses to tumors (54). However, recent improved techniques such as stereotactic body radiation therapy (SBRT) and radioembolization (RE), allow for high doses of radiation to be delivered to the tumor while limiting the damage to surrounding healthy tissues. SBRT delivers highly conformal hypo-fractionated external beam radiation in relatively fewer fractions, enabled by technological advances in on-board imaging, radiation planning, and delivery systems (12). In contrast, RE is an internal radiation technique that utilizes predominantly beta-emissions with limited range of tissue penetration from radio-labeled (e.g. Y-90) micro-embolic particles that are directly introduced to HCC *via* hepatic artery (10). These advancements have broadened the range of RT applications for HCC treatment.

SBRT has demonstrated good tumor control with 2-year local control rates between 84 and 95% (55, 56). However, the overall survial (OS) is limited by “out-of-field” intra- and extra-hepatic disease progression (57), highlighting the need for concurrent systemic disease control. SBRT is also generally well tolerated particularly in those with Child-Pugh (CP) scores less than 7. However, in those with CP score >7, there is a higher risk of radiation-induced liver disease (RILD) and worsening of CP scores (58, 59). In this group of patients, the risk of liver toxicities can be as high as 31–35%. Furthermore, SBRT may also result in gastrointestinal perforation or hemorrhage, particularly in lesions adjacent to luminal organs or those with a history of gastrointestinal ulcers (56, 60). The selective use of RE in the treatment of Barcelona Clinic Liver Cancer (BCLC) B and C HCCs have also demonstrated clinical benefit and/or local tumor control (12, 61), and less than 9% of patients who underwent RE experienced adverse events greater than grade 3 (62). While it was reported across various studies that the 20–70% of patients experienced post-RE syndrome (PRS), which included fatigue, nausea, and abdominal pain, these symptoms could be usually treated with over-the-counter drugs (62). Due to their overall efficacy and safety profile, these radiation therapies are now more widely adopted for local tumor control and bridging/downstaging treatment for future curative treatments such as transplant/resection (54, 61, 63).

**Figure 2:**
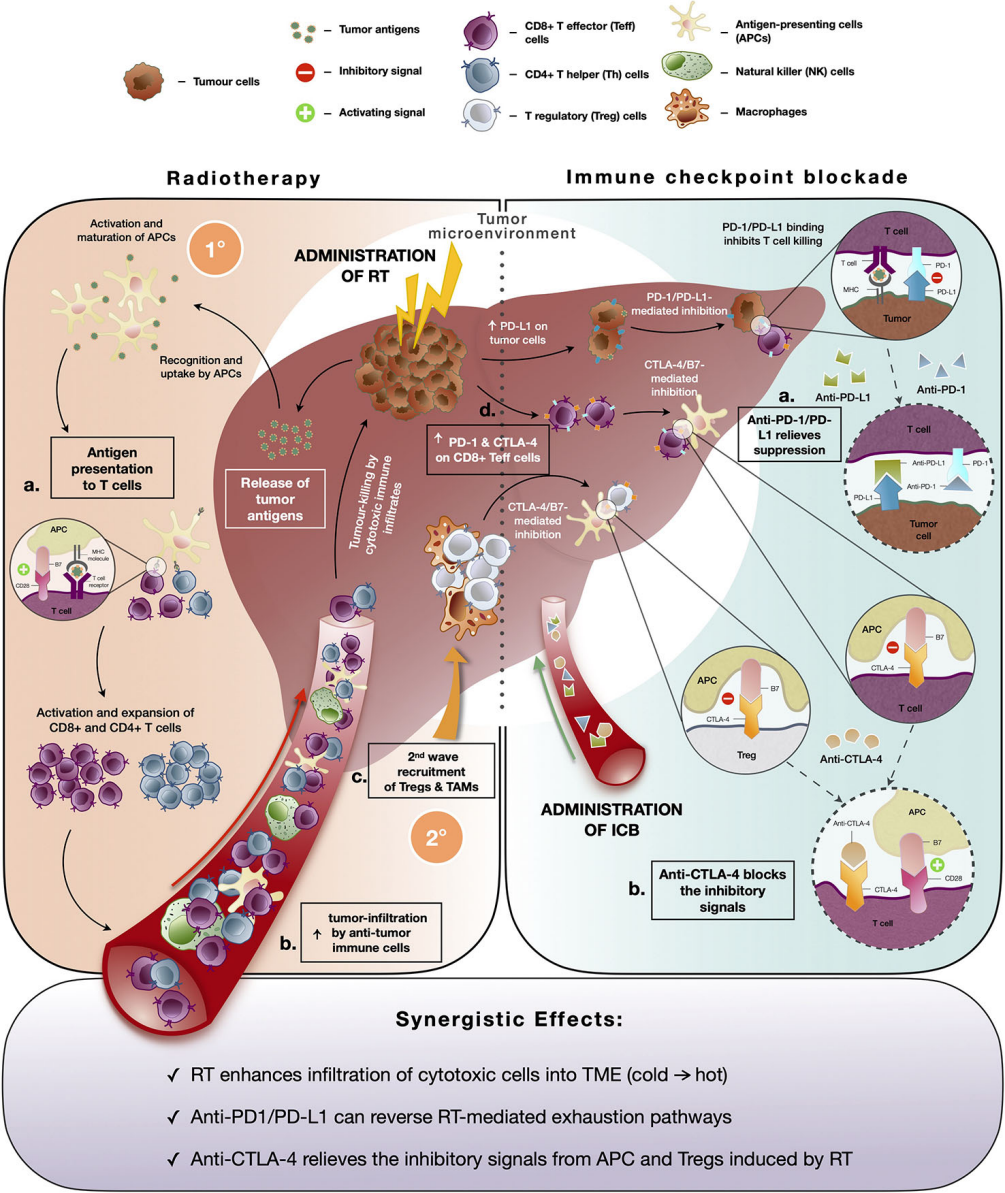
Immune modifying effects of radiotherapy (RT) and immune-checkpoint blockade (ICB). (Left panel) Immune responses induced by radiotherapy (RT): Initial (1°C) anti-tumor immune response includes **(A)** increased pool of tumor antigens and DAMPs which results in antigen presentation activity, **(B)** subsequent activation of T cells and enhanced infiltration of anti-tumor immune cells into the TME to facilitate tumor-killing; Secondary (2°C) wave of immunosuppressive response is denoted by **(C)** recruitment of immunosuppressive immune subsets, Tregs and TAMs and **(D)** upregulated expression of immune checkpoint molecules by tumor cells (PD-L1) and cytotoxic CD8+ T cells (PD-1 and CTLA-4) which dampens anti-tumor activity. (Right panel) Immune responses mediated by immune checkpoint blockade (ICB): **(A)** Anti-PD-1 and anti-PD-L1 interferes with PD-1/PD-L1 interaction and relieves suppression of CD8+ T cells by tumor cells. **(B)** Anti-CTLA-4 blocks inhibitory signaling by inhibiting B7/CTLA-4 binding and allows for the activation of APCs and T cells. (Bottom panel) Potential synergistic effects of combining RT and ICB include enhanced infiltration of anti-tumor into TME post-RT and reversion of radiation-induced exhaustion and immunosuppression by ICB.

Traditionally, the rationale behind RT for cancer treatment is to induce lethal DNA damage to tumor cells with high-energy particles leading to subsequent cell death (64). However, the ability of RT to elicit an immune-mediated anti-tumor response, a phenomenon known as “abscopal effect” denoted by the downsizing of non-targeted distant tumors following ionizing radiation treatment, has gained increased prominence in the recent decade (65). RT causes immunogenic cell death and cellular stress, which increases the pool of tumor-associated antigens and damage-associated molecular patterns (DAMPs) (66). These in turn activate dendritic cells, a professional antigen-presenting cell (APC) that primes tumor-specific CD8+ T cells to further enhance the anti-tumor responses and promote immune cell infiltration into the TME (**Figure 2**) (23). Indeed, studies revealed increased antigen presentation activity following Y-90-RE in HCC and SBRT across cancer types (22, 64). Additionally, DAMPs are taken up by PRRs which activates stimulator of interferon genes (STING) pathway that mediates the production of type I interferons (IFN-α and IFN-β) involved in the activation of downstream immune responses (67). Further evidences of radiation-induced immune responses were reported by both pre-clinical and clinical studies where heightened activation and recruitment of anti-tumor immune subsets such as CD4+, CD8+ T cells, cytotoxic NK, and CD8+CD56+ natural killer T (NKT) cells to the TME were observed (22, 64, 68). Altogether, these findings are concordant with the notion that RT can convert an otherwise “cold” tumor that has low immunogenicity and poorly infiltrated with immune cells to an immune-reactive “hot” tumor, which is well infiltrated by the immune cells (**Figure 2** **)**.

Despite the initial immune activation, RT can indirectly lead to subsequent immunosuppressive effects such as the second wave of recruitment of Tregs and TAMs to the TME (69, 70). Radiation-induced interferon activity could also cause upregulation of exhaustion molecules or signaling pathways on tumor-infiltrating cytotoxic T cells (71, 72). Our previous findings demonstrated an increase in exhausted CD8 T cells, denoted by the expression of Tim-3 and PD-1, following treatment with Y-90-RE in HCC patients (22). Other studies have also observed that RT resulted in upregulation of PD-L1 expression by the tumor cells, which can attenuate anti-tumor responses (**Figure 2**) (73, 74). Hence, tumor cells are able to subvert immunosurveillance, where immunosuppressive responses overwhelm the anti-tumor immune response and eventually result in radio-resistance. Therefore, the strategic combinational use of immunotherapy would be necessary to circumvent such resistance and to enhance clinical benefits of RT.

## Immune Checkpoint Blockades in HCC

In the past decade, the importance of immune system in tumor progression, and concept of immune-surveillance and evasion by cancer cells have been widely accepted as one of the key hallmarks of cancer (29). Immunotherapies have since gained recognition as a promising alternative cancer treatment with ICB in the forefront of clinically approved immune-modulating agents across cancer types (75). The benefits of immunotherapy in HCC have also been discussed substantially in several reviews (76–79). In particular with ICB, inhibitors against PD-1, PD-L1, and CTLA-4 have also been tested extensively in clinical trials for HCC.

**Figure 3:**
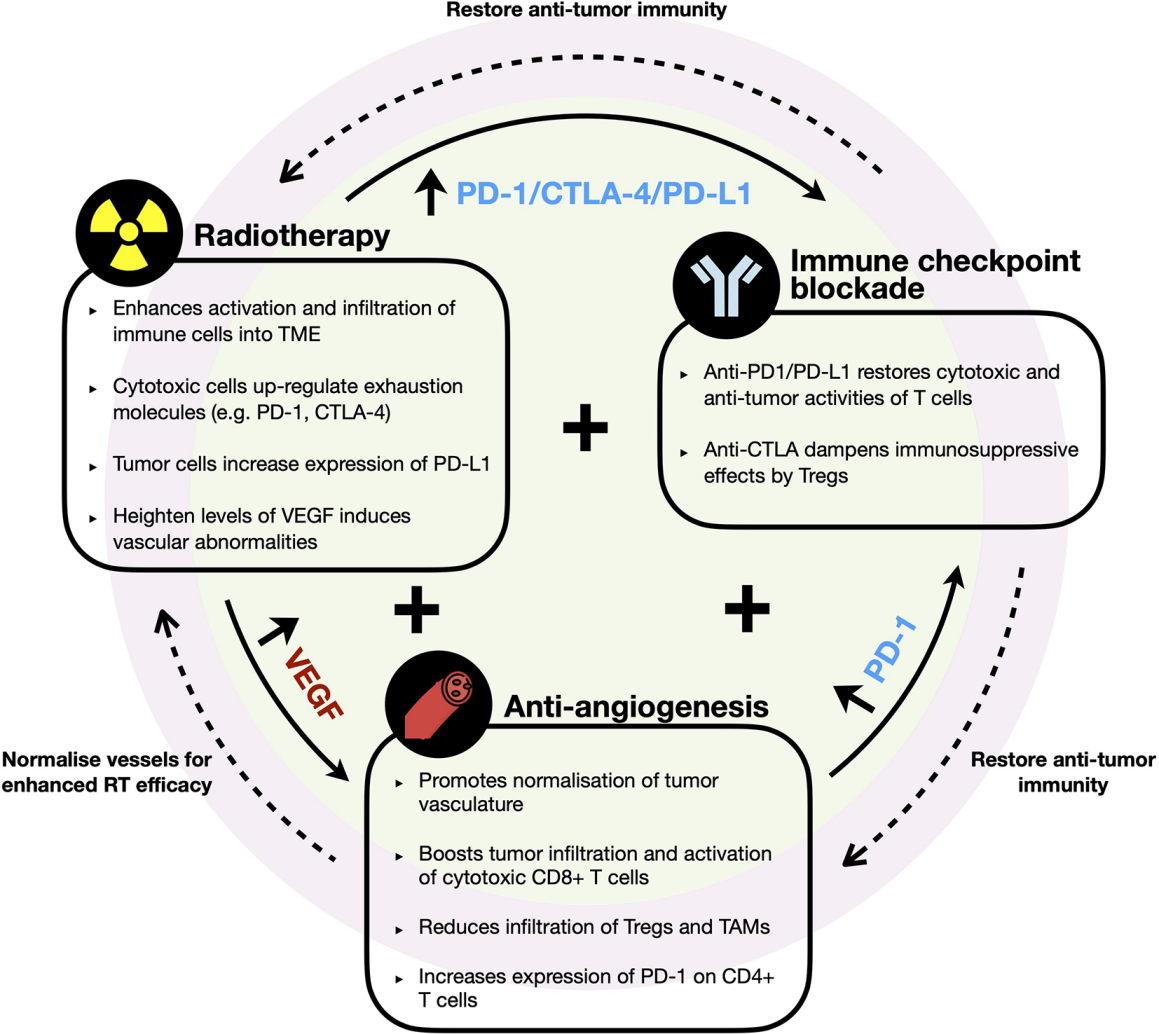
Rationale of combination therapy with radiotherapy, immune checkpoint blockade, and anti-angiogenesis agents. Diagram illustrates the key immune modifying effects by each therapeutic agent and the potential synergetic effects of their combination in HCC. RT enhances immune infiltrates into TME but induces upregulation of immune checkpoint molecules (e.g. PD-1, PD-L1, and CTLA-4) and VEGF. Anti-VEGF promotes normalization of vessel formation, which improves efficacy of RT and/or further boosts infiltration of cytotoxic cells into TME but increases PD-1 expression by CD4+ T cells. Synergistic combination with ICB restores and further enhances anti-tumor immune responses to improve efficacy of RT and anti-VEGF therapies.

Upregulation of PD-L1 expression on tumor cells, which binds particularly to PD-1 expressed by tumor-infiltrating activated T cells, induces exhaustion and dampens the anti-tumor immune activities of these effector cells hence, allowing immune evasion by tumor cells (**Figure 3** **)** (80). Inhibition of PD-1/PD-L1 interaction can reverse the exhaustive state of these cytotoxic immune cells and reinvigorate their anti-tumor activities (**Figure 2**) (81). Initial promising results were demonstrated by successful phase II ICB trials in HCC (CheckMate 040 and KEYNOTE-224) using antibodies against PD-1 (19, 20). However, subsequent phase III clinical trials (CheckMate 459 and KEYNOTE-240) which compared anti-PD-1 antibodies to sorafenib in HCC as first-line and second-line therapy, respectively, did not meet the pre-defined statistical significance improvement for OS (17, 18). Despite that, the clinical benefit of the ICB in advanced HCC is notable with superior overall response rate (ORR) and fewer incidences of grade 3/4 treatment-related adverse events in both trials (17, 18).

Another target of ICB is CTLA-4, an inhibitory receptor found on surface of T cells, which negatively modulates T cell activation and proliferation upon binding with B7 costimulatory molecule on APCs (**Figure 2**) (82). Unlike PD-1/PD-L1 pathway, CTLA-4 act primarily upstream at T cells priming stage and is also known to have a wider adverse events profile due to this (83). Both pre-clinical and clinical studies have shown that administration of anti-CTLA-4 led to the direct induction of CD4+ and CD8+ effector T cells by alleviating the immunosuppressive effect of Tregs (81, 84). Multiple phase III clinical trials investigating the use of anti-CTLA drugs, such as tremelimumab and ipilimumab, as monotherapy or in combination with other ICBs are currently underway (19, 85), with a recent FDA’s approval for the combination of ipilimumab (anti-CTLA-4) and nivolumab (anti-PD-1) in sorafenib-experienced advanced HCC patients (19).

Despite initial success observed with ICB therapies across a broad range of tumors, a decrease in their efficacy and acquired resistance were reported following initial response to ICB (86, 87). This varying response rate in HCC patients have been intensively reviewed previously (88–90). Despite a previous study reporting the distinct immune landscape between viral *versus* non-viral HCC (30), there is no concrete evidence that suggests a difference in response rate between viral *versus* uninfected HCC towards ICBs to date. Patients from various cancer types with higher tumor PD-1 and PD-L1 expression were evidenced to have improved OS and response as compared to those with lower expression levels (86). However, PD-L1 expression was not a significant biomarker for response to anti-PD-1 in HCC as evidenced by the findings from ICB trials CheckMate 040 and KEYNOTE-224 (20, 91). Studies have also attributed the innate variability of each patient’s pre-existing immunity to the differential response observed towards various ICB therapies across cancer types (92–94). Specifically in HCC, tumors with higher transcriptomic diversity were associated with worse OS in patients treated with ICB and these tumor cells also expressed a significantly higher level of vascular endothelial growth factor A (VEGF-A) (95). Concordant to this finding, Chen et al. showed in a separate study that responders to anti-PD-1 treatment have decreased expression levels of VEGF-A while the non-responders have increased VEFG-A expression (94). This in turn suggests that the VEGF pathway is an important mechanism for resistance to the anti-PD-1 therapy, where it could hamper tumor infiltration and functions of T effector cells (96, 97). Therefore, the degree of transcriptomic diversity of HCC tumors, VEGF expression level, and pre-existing immunity of each individual could provide a rationale for the observed differential responses of HCC patients towards immune checkpoint blockade therapies.

## Combinational Strategies: RT AND ICB

The interest in combining radiotherapy and immunotherapy stems from the rationale that radiation primes the immune system and produce a synergistic anti-tumor immunity for durable disease control when combined with immunotherapy (**Figure 2**, bottom). For instance, RT enhances inflammatory immune response and intra-tumoral infiltration by cytotoxic immune cells while ICB could overcome the radiation-induced exhaustion in CD8 T cells and restore their anti-tumor immune responses. Kim et al. demonstrated the dose-dependent upregulation of PD-L1 expression following irradiation of various HCC cell lines, which was found to be mediated predominantly through the IFN-γ-STAT3 signaling pathway (25). Likewise, clinical studies have reported post-RT upregulated expressions of PD-1 and PD-L1 by CD8 T cells and tumor cells, respectively (22, 73, 74). These studies provided a sound rationale for the combination of RT with ICB, which should be considered against the major concerns for a successful treatment strategy in HCC as outlined in **Table 1**.

**Table 1:** Major considerations for the combination of RT and ICB.

Major concerns of intervention(s)	Potential solution
How can we subvert RT-induced exhaustion?	ICB that targets exhaustion pathways can help reinvigorate the exhausted cytotoxic immune cells (24, 26, 73, 74).
How to overcome the ineffectiveness of ICB against cold tumors?	RT can trigger immune activity and switch a “cold” tumor to a “hot” tumor with enhanced inflammation and tumor infiltration by the immune cells (22, 25, 64, 68).
Will there be severe toxicities in the combined therapy of RT and ICB?	Preliminary findings from early phase trials for combined use of RT and ICB have showed tolerable safety profile (98–100).
How can we predict the differential responses by patients towards treatments?	Discovery of predictive biomarkers for response towards various cancer treatments (i.e. RT and ICB) and in combination is essential to select the most appropriate therapeutic agent and therapy for the patients (22, 95, 101).

Preclinical data have shown that the combination of RT and ICB exhibited therapeutic synergism as well as superior tumor control (24–26, 73). Significant tumor growth suppression and improved OS were observed in HCC mice treated with single 10Gy RT and anti-PD-L1 compared to either therapy alone (25). Anti-PD-L1 and 12Gy RT exerted abscopal tumor control and superior local control of irradiated tumors in a mammary cancer murine xenograft model compared to monotherapy (24). Similar findings were replicated in CT26 murine colon carcinoma xenografts treated with fractionated RT (2Gy x 5) and/or anti-PD-1 (73). Synergism between RT and anti-CTLA-4 were also demonstrated in several murine tumor models (26, 102). More importantly, these effects were durable as shown by the rejection of tumor re-challenge that was dependent on CD8 T-cells (24, 73).

Despite encouraging preclinical findings, there is no published prospective clinical data to our knowledge on combined RT and ICB therapy in HCC, except a few small series that have shown promising clinical activity (98, 99). Chiang et al. reported 100% ORR in 5 patients treated with SBRT followed by nivolumab for large unresectable HCC and another case report showed complete pathological response following Y-90-RE and nivolumab bridging therapy prior to partial hepatectomy (98, 99). Tai et al. also showed that combination of Y-90-RE with nivolumab had an optimistic ORR of 31%, and the combination therapy was safe and tolerable with only 11% of treated advanced HCC patients experienced grade 3/4 treatment-related adverse events (100). Phase I trial (NCT02239900) that evaluated liver/lung SBRT with ipilimumab reported that 23% of patients experienced clinical benefit which corresponded to increase in CD8+ T cells and CD8+/CD4+ ratios (101). Grade 3 toxicities were found in 34% of patients with no grade 4/5 toxicities. A separate phase I basket trial evaluated the safety of multi-site SBRT followed by pembrolizumab in patients with advanced solid tumors (NCT02608385), including one case of HCC. Similar levels of toxicity were observed as compared to monotherapy-treated patients with a dose-limiting toxicity rate of 9.7% (103). Several other prospective clinical trials are currently on-going to evaluate the combined approach of RT and immunotherapy in HCC (**Table 2**).

**Table 2:** Ongoing clinical trials investigating combined use of RT and ICB in HCC/liver cancer.

Clinical trial identification(Study Name)	Phase	Disease	Type of radiative intervention	Type of ICB	Treatment design	Est. enrolment	Primary endpoint	Secondary endpoints
NCT03033446	II	HCC	Y-90 RE	Nivolumab	Y-90 RE -> Nivolumab	40	RR	TTR, DoR, TTP, PFS, OS, QOL, AEs
NCT03482102	II	HCC and biliary tract cancer	Radiotherapy (not specified)	Durvalumab(anti-PD-L1) and Tremelimumab (anti-CTLA_4)	Durvalumab + tremelimumab -> RT	70	Best ORR	AEs, OS, DCR, PFS, DoR, TTP
NCT02239900	II	Liver and lung cancer	SBRT	Ipilimumab(anti-CTLA-4)	Ipilimumab + SBRT orIpilimumab -> SBRT	143	MTD	RR
NCT02608385	I	Solid tumors	SBRT	Pembrolizumab(anti-PD-1)	SBRT -> pembrolizumab	130	Recommended SBRT dose	AEs, long-term AEs, RR, PFS, OS, LC, radiation-induced changes in TME, DCR
NCT03203304	I	HCC	SBRT	Nivolumab or Ipilimumab	SBRT -> Nivolumab or Nivolumab + ipilimumab	50	AEs	ORR, long-term AEs, PFS, OS, DCR, LC
NCT03817736 (START-FIT)	II	HCC	TACE + SBRT	Not specified	TACE + SBRT -> ICB	33	No. of patients amendable to curative surgical intervention	RR, TTP, PFS, OS, QOL, AEs, PR

RE, radioembolization; SBRT, stereotactic body radiation therapy; TACE, transcatheter arterial chemoembolization; RR, response rate; ORR, overall response rate; MTD, maximum tolerated dose; AEs, adverse events; TTR, time to response (TTR); DoR, duration of response; TTP, time to progression; PFS, progression-free survival; OS, overall survival; QOL, quality of life; DCR, disease control rate; LC, local control; PR, pathological response; ->, followed by.

As this field is in its preliminary stages, most studies have showed encouraging efficacies and tolerable toxicity profile in patients treated with combination of RT and ICB but did not specify an optimal RT dose, fractionation scheme and RT/ICB sequencing. There is consensus that these parameters are highly dependent on cancer type, choice of ICB, as well as tumor histology and mutational burden (104). In addition, hypo-fractionation appears to be favored over conventional fractionation as it appears to elicit a more effective anti-tumor response and a dose of 8–10Gy RT with one to three fractions was suggested to induce abscopal effect (104, 105). Considerations for the radio-sensitivity of the surrounding vasculature, toxicity profile and identification of pro-immunogenic signatures following RT would also be essential to optimize protocols for combining RT and ICB (106). Therefore, it is imperative to understand the key immune checkpoint molecules modified by RT in the TME, the sequence and mechanisms of their modification and subsequent role in tumor immunity to determine the type of ICB to be used and RT/ICB dosing sequence.

## Future Developments in Combinational Strategies for HCC

Beyond the combination of RT and ICB, there have been remarkable advancement in the use of anti-angiogenesis agent in treatment for HCC reported by the phase III IMbrave150 trial, which demonstrated superiority in terms of ORR, OS, and PFS in HCC patients treated with atezolizumab (anti-PD-L1) and bevacizumab (anti-VEGF-A) *versus* patients treated with sorafenib (21). As one of the hallmarks of cancer, angiogenesis plays critical role in tumor formation and growth and have long been a promising drug target in HCC (29, 31). Dysregulated VEGF expression was shown to be responsible for the vascular abnormalities observed in HCC tumors (31, 107). Various other studies have also consistently showed that elevated circulating VEGF expression following surgery or RFA correlated to poor prognosis in HCC patients (31, 108, 109). Shigeta et al. revealed the mechanism behind the anti-tumorigenic effects of this anti-PD-L1 and anti-VEGF-A dual therapy using orthotopic murine HCC model. They reported that VEGFR2-blockade alone increased PD-1 expression on CD4+ cells in the tumor but when combined with anti-PD-1 therapy, CD4+ cells’ functions were restored and aided in normalization of vessel formation. The combination therapy also increased tumor infiltration and activation of cytotoxic CD8+ T cells, while reducing infiltration of Tregs and monocytes (110).

Importantly, VEGF expression was found to be elevated in post-RT treated HCC tumors (109). Based on this, we propose that it would be meaningful to examine the efficacy of treating HCC patients with anti-angiogenesis and ICB following RT due to their ability to fuel efficient tumor-killing activities and limit the tumor cells’ ability to re-stablish themselves **(** **Figure 3**). An alternative strategy would be to administer anti-angiogenic agent prior to RT, which could “normalize” tumor vasculature and in turn, potentially promote greater efficacy of the radiotherapy effects (111). ICB could be administered following this combination treatment to further sustain anti-tumor immunity **(** **Figure 3**). However, such treatment regimens have to be carefully evaluated as anti-angiogenics are associated with increased risk of severe RT-related gastrointestinal luminal toxicities (112). Other potentially more tolerable combination such as dual ICBs with RT could also be considered.

Apart from the current immune checkpoint inhibitors that have been heavily evaluated in HCC, we can also consider other novel targets such as indoleamine 2,3 dioxygenase (IDO). IDO is an enzyme that is involved in immune homeostasis and also escape from immunosurvelliance by tumor cells (113). Overexpression of IDO in HCC tumors was associated to poor prognosis where dendritic cells suppress T cells *via* IDO and contribute to progression in HCC (114). However, Ishio et al. found that IDO could also have anti-tumor properties and its expression level was correlated with gene expressions of IFN-γ, tumor necrosis factor alpha (TNFα), and interleukin 1 beta (IL-1β) in HCC (115). Another study found that increased tumor expression of IDO (T-IDO) correlated with increased CD8+ T cells infiltration and favorable outcome in HCC (116). Contrary to above findings, a study conducted in patients of NSCLC found that low activity of IDO following chemoradiation was associated favorably with survival but the effect of radiation on the activity level of IDO was heterogenous (117). A preclinical study carried out with Lewis lung mouse model also showed that treatment with IDO inhibitor and RT synergistically reduced Tregs and expression of exhaustion molecules such as PD-1, PD-L1, and TIM-3 by dendritic cells and T cells (118). Taken altogether, the potential of IDO as a combination therapy with RT in HCC remains to be determined.

Currently, several other B7 family ligands such as B7-H2 (a.k.a. inducible T cell costimulator ligand; ICOSL) and B7-H3 are being explored as novel immunotherapeutic targets in other cancer types (119). In HCC, both independent or combined expression of B7-H2 and B7-H3 have correlated to recurrence and poorer survival rate of the patients (120). Although further understanding of the tumor-promoting mechanisms by B7 ligands in the context of HCC is required, they could become promising combinatorial agents in treating HCC in the future. Similarly, chimeric antigen receptor T (CAR-T) cell therapy have shown promising results in lymphoma patients and solid tumors with strong driver mutations that could be used as specific targets for CAR-T cell (121, 122). However, HCC is a largely transcriptomically heterogeneous solid tumor (95) and attempts to target HCC-specific antigens, such as AFP, had been evaluated previously with disappointing outcomes (NCT03349255). Nonetheless, findings from a recently concluded phase I trial (NCT02395250) which investigated the safety profile of glypican-3 (GPC3) CAR-T cell therapy in HCC patients showed that it was well tolerated and demonstrated indications of anti-tumor activity (123). GPC3 is involved in the regulation of cell division and growth and its expression in tumor cells was previously associated with poor prognosis for HCC (124). The rapid development in the safety and efficacy of CAR-T cell therapies for solid tumors is optimistic and are highly anticipated as the future of novel immunotherapeutic strategies in cancer.

A crucial aspect to ensure a successful treatment outcome lies in the selection of patients who would benefit most from any given treatment strategies. Mounting evidence shows that variability in pre-existing immunity of each patient reflects their respective clinical response to immunotherapies (93, 94). Crittenden et al. also demonstrated that *in vivo* blockade of pre-existing immune responses in mice rendered the combination of RT and ICB ineffective (125). However, there are limited studies on predictive biomarkers for response in various HCC treatments to date. Initially, tumor tissue expression of PD-L1 was used to select patients for enrolment into various ICB trials, however, it was later proven not a strong predictor of response due to heterogeneity in its expression in tumor tissues and various technical issues with detection of its expression (126, 127). Instead, a recent study in a small cohort of liver cancer patients showed that higher cytolytic T cell infiltrates is associated with response towards ICB in HCC (95). Our group constructed a predictive model based on pre-treatment PBMCs of HCC patients and concluded that existence of pre-activated PD-1^+^ and Tim-3^+^CD8^+^ T cells with homing capability (CCR5^+^ and CXCR6^+^) was key in predicting response to Y-90-RE (22). Similarly, peripheral immune phenotypes may predict for therapeutic efficacy of SBRT and concurrent SBRT and ICB therapy (101, 128). The use of radio-sensitivity index and radiosensitive gene signatures have also been heavily investigated but fell short in their value as predictive biomarkers (129). Taken altogether, the lack of reliable predictive biomarker(s) for various treatments in HCC indicates that greater effort and emphasis is required in this area in order to more accurately select for therapy that provides the maximum clinical benefit and minimal adverse effect for patients.

## Concluding Remarks

The immunosuppressive landscape of HCC, as evidenced by the presence of pro-tumoral and exhausted immune cells, renders ICB a promising treatment option for HCC patients. Improvements in radiation techniques over the past two decades have boosted the efficacies of RT in HCC. In combination, ICB could overcome the upregulation of immune checkpoint molecules/pathways induced by RT and restore the anti-tumor immunity. In turn, RT can also re-model an otherwise “cold” TME to an immune-reactive “hot” TME, which synergistically improves the effectiveness of ICB. Beyond RT and ICB, combinational use of anti-angiogenesis agent, which normalizes tumor vasculature and induce local immune response in TME, can also be explored. Most importantly, the sharp increase in the variety of combinational immunotherapeutic options in recent years demands for the discovery of predictive biomarkers in order to determine the most appropriate therapeutic strategy for optimal clinical benefit.

## Author Contributions

YL and VC contributed in design, drafting, revising, and approving the final version of the manuscript. DT, CY, and SC assisted in editing the manuscript. All authors contributed to the article and approved the submitted version.

## Funding

This work was supported by the National Medical Research Council (NMRC), Singapore (ref numbers: TCR15Jun006, CIRG16may048, CSAS16Nov006, CSASI17may003, and LCG17MAY003).

## Conflict of Interest

The authors declare that the research was conducted in the absence of any commercial or financial relationships that could be construed as a potential conflict of interest.
